# Convolutional neural network for efficient estimation of regional brain strains

**DOI:** 10.1038/s41598-019-53551-1

**Published:** 2019-11-22

**Authors:** Shaoju Wu, Wei Zhao, Kianoosh Ghazi, Songbai Ji

**Affiliations:** 1 Department of Biomedical Engineering, Worcester Polytechnic Institute, Worcester, MA USA; 2 Department of Mechanical Engineering, Worcester Polytechnic Institute, Worcester, MA USA

**Keywords:** Engineering, Biomedical engineering

## Abstract

Head injury models are important tools to study concussion biomechanics but are impractical for real-world use because they are too slow. Here, we develop a convolutional neural network (CNN) to estimate regional brain strains instantly and accurately by conceptualizing head rotational velocity profiles as two-dimensional images for input. We use two impact datasets with augmentation to investigate the CNN prediction performances with a variety of training-testing configurations. Three strain measures are considered, including maximum principal strain (MPS) of the whole brain, MPS of the corpus callosum, and fiber strain of the corpus callosum. The CNN is further tested using an independent impact dataset (N = 314) measured in American football. Based on 2592 training samples, it achieves a testing *R*^2^ of 0.916 and root mean squared error (RMSE) of 0.014 for MPS of the whole brain. Combining all impact-strain response data available (N = 3069), the CNN achieves an *R*^2^ of 0.966 and RMSE of 0.013 in a 10-fold cross-validation. This technique may enable a clinical diagnostic capability to a sophisticated head injury model, such as facilitating head impact sensors in concussion detection *via* a mobile device. In addition, it may transform current acceleration-based injury studies into focusing on regional brain strains. The trained CNN is publicly available along with associated code and examples at https://github.com/Jilab-biomechanics/CNN-brain-strains. They will be updated as needed in the future.

## Introduction

Traumatic brain injury (TBI) remains a major public health problem in the world^1,2^. According to the ﻿World Health Organization, more than 40 million people worldwide suffer from a mild TBI (mTBI) each year^3^. In the United States alone, the number of concussion incidents could reach 1.6–3.8 million annually, and is particularly common in athletes playing contact sports^4,5^. Although mild in nature, about 300,000 of the incidents involve loss of consciousness, with the majority occurring in American football^6^.

To mitigate the risk of concussion, head impact sensors such as Head Impact Telemetry (HIT) System^7^ and mouthguards^8,9^ are deployed in many contact sports. They record impact kinematics upon head collision and have been extensively used to measure head impact exposure^10^. However, only peak linear and/or rotational accelerations such as “g-forces” are often used that do not inform impact-induced brain strains thought responsible for brain injury^11,12^. Consequently, there is question about their effectiveness in concussion detection^13^.

Using measured kinematics as input, a sophisticated computational head injury model can estimate detailed brain strains. They are generally believed to be more effective than impact kinematics in detecting brain injury, including concussion^14^. However, a significant challenge preventing injury models from real-world use such as facilitating head impact sensors for concussion detection on the sports field is that they are too slow—typically requiring hours to simulate even a single head impact on a high-end workstation^15–17^. As a result, the use of head injury models has been largely restricted to retrospective research efforts to date with no obvious clinical diagnostic value, and head impact sensors are also significantly underutilized.

There exist two competing strategies to mitigate the computational cost in model simulation. They share similarities in conceptualizing a head injury model as a nonlinear, high-dimensional, but smooth and continuous mapping function between impact kinematics and brain responses^15,18^. One strategy is to simplify the model and response output. For example, several reduced-order models have been proposed, including a one-degree-of-freedom (DOF) dynamic model based on modal analysis^19^ or equation of motion^20^, and a second-order model^21^. By fitting parameters of a reduced-order model against directly simulated responses obtained from a finite element (FE) model of the human head, hybrid brain injury metrics were developed to correlate with peak maximum principal strain (MPS) of the whole brain. These metrics have shown promise over other conventional injury metrics derived solely from rotational kinematics when correlating against MPS of the whole brain over a large spectrum of impact severities and in diverse injury scenarios on a group-wise basis^21,22^.

However, MPS estimation accuracy from reduced models degrade for large strain impacts^21,22^, presumably as a result of failing to capture the significant nonlinearities when the impact severity is high. Unfortunately, large strains from more severe impacts (vs. low severity blows) likely would be the most important to consider when assessing the risk of brain injury on an individual basis. Further, reduced models relying on MPS of the whole brain lose critical information on brain strain distribution, because the strain variable is overly simplified to a single scalar value for the entire brain and is not region-specific. It is also unclear how reduced models could estimate directionally informed “axonal”^23–25^ or “fiber”^26^ strains that characterize stretches along white matter fiber tracts, or strain rate^27^ thought important to brain injury as well. These observations indicate some inherent limitations with reduced-order models in practical applications, despite their potential for gaining physical insight into the induced brain strains.

To preserve the nonlinearity and spatial distribution of brain strains, a pre-computed brain response atlas was also introduced^15^. Instead of simplifying the model and output, this approach idealizes impact kinematic profiles serving as model input. Brain strains are pre-computed for a large library of impacts by discretizing peak rotational acceleration/velocity, and azimuth and elevation angles of head rotation^28^. Element-wise MPS values (vs. peak magnitude of the whole brain^21,22^) for an arbitrary impact are then interpolated/extrapolated instantly. The pre-computed response atlas was shown to be effective using dummy head impacts simulating American football^28^. However, the idealized rotational kinematic profiles are limited to triangular shapes of acceleration impulses that do not include deceleration. For more complex kinematic profiles involving deceleration and velocity reversal, the atlas may need to be expanded to include additional characteristic impacts. Unfortunately, this requires an explicit analysis of rotational velocity profile shapes, which is not trivial^28,29^.

Instead of simplifying the impact kinematic input^15,28^, head injury model, or response output^19–21^, here we develop a convolutional neural network (CNN) to learn the nonlinear impact-strain relationship without any simplification. The CNN is a typical data-driven approach where the network weights are determined by the given training data iteratively *via* backpropagation. By “implicitly” capturing important features of head rotational velocity profiles, regional brain strains can be estimated instantly with sufficient accuracy while maintaining the fidelity of impact kinematics, the sophistication of a head injury model, and the detailed response output. Such a capability is critical for effective real-world applications such as concussion detection on the sports field.

CNN is a class of deep learning neural network that has been extensively used in medical imaging and computer vision^30,31^. Object detection and pattern recognition are achieved *via* local filter convolution to capture structural information among neighboring pixels/voxels^32,33^. This is analogous to detecting local shape variations in head rotational kinematic temporal profiles that serve as model input to determine brain strains. This inspired us to conceptualize time-varying biomechanical signals of head rotation, specifically, rotational velocity profiles along the three anatomical directions, as two-dimensional (2D) images to apply CNN for response regression. The image representation preserves the temporal locality of head rotational velocity as the three components along the temporal dimension are given at the same time. In contrast, concatenating the three velocity profiles into a one-dimensional (1D) vector may not work well with existing CNN architectures because it destroys the temporal locality required for data convolution.

We organized the study as the following. We first used two real-world impact datasets to generate sufficient training samples through augmentation. Next, we empirically optimized a CNN architecture and probed its testing performance behaviors under a variety of training-testing configurations. All training samples were then combined to conduct a 10-fold cross-validation^34^ and to further test on a third, independent impact dataset measured in American football^35^. Finally, an additional 10-fold cross-validation was conducted using all response samples available. The CNN technique developed here allows instantly converting a head impact measured from impact sensors into regional brain strains. Therefore, it may facilitate head impact sensors in monitoring neural health and detecting concussion more effectively; thus, transforming kinematics-based TBI studies into brain strain-related investigations in the future.

## Materials and Methods

### Data augmentation

We used two impact datasets to generate CNN training samples: (1) video-confirmed impacts measured in American college football, boxing, and mixed martial arts from Stanford University (SF; N = 110)^8^; and (2) lab-reconstructed impacts from National Football League (NFL; N = 53)^36^. The SF dataset was measured using instrumented mouthguards^8^. The latter were reconstructed and recently reanalyzed^36^ in the laboratory using dummy heads by matching the location, direction, and speed of the impacts approximated from video analysis^37^. As contribution of linear acceleration to brain strains was negligible^38^, only isolated 3-DOF rotational velocity profiles in a ground-fixed coordinate^28^ were used. The data sizes were small compared to typical CNN applications (e.g., thousands or even millions^33^). Therefore, data augmentation was necessary to increase the variation in head rotational kinematics for training.

**Figure 1:**
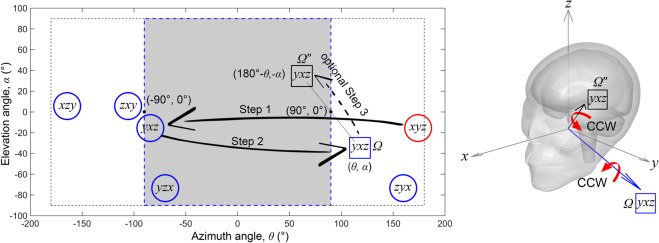
Illustration of data augmentation for a representative head impact rotational profile in terms of rotational axis, Ω(*θ*,*α*). Rotational velocity components along the three anatomical axes were permuted (Step 1), randomly rotated (Step 2), and then converted to its conjugate rotational axis, Ω″ (optional Step 3), if the azimuth angle, *θ*, is outside the sampling range (shaded area). Counter clockwise (CCW) rotation about Ω″ generates a mirroring response about the mid-sagittal plane relative to CCW rotation about Ω^15^.

To do so, components along the *x*, *y*, and *z* directions were permuted to construct 6 (*N* = 3!) *v*_*rot*_ profiles (i.e., *xyz*, *xzy*, *yxz*, *yzx*, *zxy*, and *zyx*; Step 1 in Fig. 1). Each profile was further rotated about a random axis passing through the head center of gravity with a random magnitude (within 0–90^0^; Step 2 in Fig. 1)^28^. The azimuth and elevation angles (*θ* and *α*, respectively) of the rotational axis, Ω(*θ*,*α*), were then determined based on peak magnitude of rotational velocity^28^. Due to head symmetry about the mid-sagittal plane, only half of the Ω sampling space was necessary (shaded area in Fig. 1)^15^. Therefore, for Ω with $\Vert \theta \Vert  > 90^\circ$, its corresponding “conjugate rotational axis”, Ω″(180°−*θ*, −*α*), was used to maximize the use of *v*_*rot*_ profiles for generating unique brain responses (optional Step 3 in Fig. 1).

Finally, to focus on more “severe” head impacts relevant to potential “injury”, all profile magnitudes were randomly scaled so that the peak resultant velocity magnitude was above the median concussive value of 21.9 rad/s found in football while below 40 rad/s, sufficient to capture the 95^th^ percentile of 34.1 rad/s^39^. For a given impact dataset, each permutation and random perturbation/scaling constituted one batch of training data.

The augmented SF dataset was used to optimize a CNN architecture, including the number of layers and their associated parameters (details below). We found two batches of *v*_*rot*_ profiles (N = 1320, 110 × 6 × 2) were necessary to yield a coefficient of determination (*R*^2^) above 0.90 (deemed successful) in a 10-fold cross-validation^34^. Similarly, we generated two batches of *v*_*rot*_ profiles for the NFL impacts (N = 636, 53 × 6 × 2). Some impacts from the augmented datasets may not be physically admissible or rare to occur; still, they uniquely probed the impact-strain response hypersurface and were useful for CNN training.

### Data preprocessing

**Figure 2:**
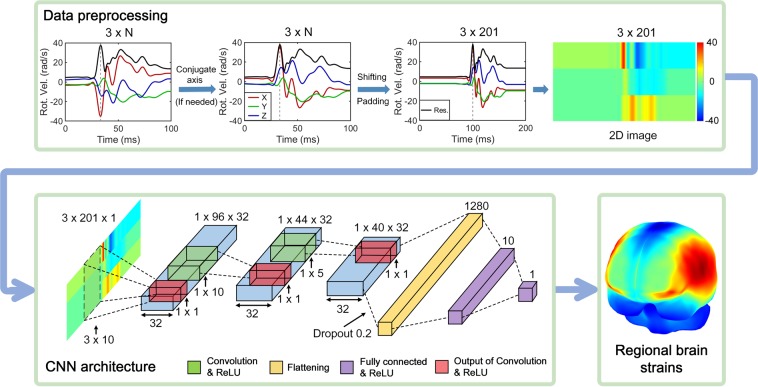
Overview of impact data preprocessing (top) and an empirically optimized CNN architecture (bottom) for training and testing. A typical rotational velocity temporal profile is conceptualized as a 2D image for CNN input. Note the difference in velocity profile temporal axes, as no curves are “squeezed”. The technique is illustrated for three strain variables in this study but can be easily extended to the whole-brain, element-wise responses. CNN: convolutional neural network.

The CNN requires a fixed input size. Therefore, all *v*_*rot*_ profiles were reformatted into a 3 × 201 matrix. The first dimension was fixed to represent time-varying *v*_*rot*_ components along the three anatomical directions. The second dimension corresponded to 200 ms in temporal length at a resolution of 1 ms (from 0 to 200 ms; sufficient for all impacts used). The second dimension could be adjusted based on the impact temporal resolution, which may require adjusting the CNN filter and stride sizes accordingly (details below). As peak rotational velocity was known to be important for brain strains, we synchronously shifted the three *v*_*rot*_ components so that the resultant peak occurred at a fixed time point of 100 ms (Fig. 2). At both ends of the rotational velocity profile, replicated padding was used to maintain a zero acceleration, where values at the two velocity profile borders were replicated along the temporal axis. Controlling the temporal location of the resultant velocity peak reduced kinematic data variation, which was expected to decrease the number of training samples required because the same shifting and padding were applied to all testing *v*_*rot*_ profiles. Repositioning *v*_*rot*_ profiles in time did not affect output, as the strain responses were accumulated maximum values, regardless of the time of occurrence.

### Impact simulation

All impacts were simulated using the recent Worcester Head Injury Model (WHIM) that incorporates anisotropic material properties of the white matter based on whole-brain tractography^40^. The anisotropic WHIM employs the same mesh and brain-skull boundary conditions as in the previous isotropic version^26^. It was successfully validated against six cadaveric impacts and an *in vivo* head rotation^40^.

Three strains from model simulations were separately used for training and testing: MPS of the whole brain, MPS of the corpus callosum (CC; a particularly vulnerable region^41,42^), and fiber strain of the CC, all assessed at the 95^th^ percentile level. Their increasing level of sophistication (whole brain vs. region-specific; direction invariant MPS vs. directionally informed fiber strain) was designed to stress test the CNN prediction capability.

### CNN architecture

Application of CNN in TBI investigation is limited to image-based tasks so far, such as mimicking neuronal behaviors in cognitive deficits^43^ and contusion image segmentation^44^. Its application in biomechanics has also emerged, for example, to predict musculoskeletal forces^45^ and to perform mobile gait analysis^46^. However, application of CNN in TBI biomechanics does not yet appear to exist.

Therefore, we first empirically optimized a CNN architecture^45^ using whole-brain MPS obtained from the augmented SF dataset. The number of CNN filters and their sizes and stride sizes were iteratively and empirically updated until a 10-fold cross-validation performance was maximized in terms of *R*^2^ between the predicted and directly simulated responses. Figure 2 shows the optimized CNN, which led to a maximized *R*^2^ of 0.937 with root mean squared error (RMSE) of 0.018. The 32 filters had sizes of 3 × 10, 1 × 10 and 1 × 5 with stride sizes of 1 × 2, 1 × 2 and 1 × 1 for the three convolutional layers, respectively. They were followed by a flattening layer (with a dropout rate of 0.2^47^) and two fully connected layers. Rectified linear unit (ReLU) activation functions^48^ were used.

The same optimized CNN architecture was employed for all subsequent trainings with 250 epochs, an initial learning rate of 10^−6^, and a batch size of 64. Mean squared error (MSE) between the predicted and directly simulated strain measure of interest served as the loss function for minimization *via* an adaptive moment estimation (Adam) optimizer^49^ implemented in Keras (Version 2.08)^50^. Validation-based early stopping^51^ was used to avoid overfitting.

### Performance evaluations

All testing *v*_*rot*_ profiles followed the same preprocessing steps (Fig. 2). We first used the augmented SF dataset to train and test on the reconstructed NFL dataset, and conversely, used the augmented NFL dataset to train and test on the measured SF dataset. To probe the importance of training sample size on testing performances, two additional batches were generated for the augmented NFL dataset, leading to a total of 1272 *v*_*rot*_ impacts (53 × 6 × 4; comparable to the augmented SF data size). We then repeated the same training and testing on the measured SF dataset. To further investigate the importance of *v*_*rot*_ profile shape characteristics in training (measured on-field vs. lab-reconstructed), we also used the augmented SF dataset to train and estimate strains in the measured SF dataset, as they were from the same data source and shared the same *v*_*rot*_ resultant profile shapes on a group-wise basis. For completeness, the augmented NFL dataset was also used to train and test on the reconstructed NFL impacts.

Next, we combined the augmented SF and NFL datasets (N = 2592) and conducted a 10-fold cross-validation. To demonstrate real-world use of our technique, we used the combined dataset to train and test on a third, independent impact dataset measured and video-confirmed using an impact monitoring mouthguard^9^ in American high school football (HF; N = 314^35^). Finally, all impact-strain response samples available in this study were combined (N = 3069; 1320 + 1272 + 110 + 53 + 314) to conduct a final 10-fold cross-validation.

For each strain measure, we evaluated testing accuracy using *R*^2^ and RMSE. Because the augmented datasets intentionally focused on more severe head impacts most relevant to injury, we reported the testing performances for impacts within the focused rotational velocity peak range, in addition to the full dataset.

### Statistical tests

For all tests, 30 trials with random CNN initialization seeds were conducted (*n* = 30). For 10-fold cross-validations, their performances were compared using a corrected one-tailed *t*-test to avoid high Type I error^52^. For other tests, a Welch’s one-tailed *t*-test was used to account for unequal variances^53^. Significance level was set at 0.05.

### Data analysis

Although all velocity profiles were reformatted to 200 ms as CNN input, only impact profiles prior to shifting and padding were necessary for simulation (Fig. 2), as the padded zero accelerations had no effect on peak brain strains. Each impact of 100 ms required ~30 min for simulation with Abaqus/Explicit (double precision with 15 CPUs and GPU acceleration; Intel Xeon E5-2698 with 256 GB memory, and 4 NVidia Tesla K80 GPUs with 12 GB memory) and another ~30 min for post-processing to calculate regional strains. In total, 3069 impacts were simulated, typically with 5–10 jobs running simultaneously. Training a CNN required ~3 min per fold on an NVIDIA Titan X Pascal GPU with 12 GB memory, while predicting on a testing profile was instant (<0.1 sec) on a low-end laptop. All data analyses were conducted in MATLAB (R2018b; MathWorks, Natick, MA) and Python (Version 2.7.0).

## Results

### Performance evaluation: between the two datasets

**Figure 3:**
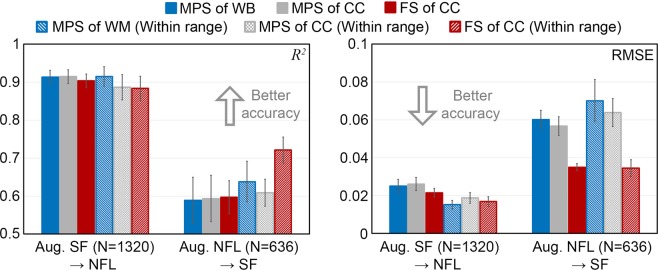
Average *R*^2^ (left) and RMSE (right) from 30 random trials (with bars indicating standard deviation) using augmented SF dataset to predict responses in the reconstructed NFL impacts, and vice versa. For each strain measure, performances are reported using either impacts from the entire dataset or those within the targeted resultant rotational velocity peak range (within-range). MPS: maximum principal strain; FS: fiber strain; WB: whole brain; CC: corpus callosum.

Using the augmented SF dataset for training and testing on the reconstructed NFL impacts consistently achieved significantly higher *R*^2^ and lower RMSE than the other way around switching the two datasets for training/testing for the same strain measure (*p* < 0.001; Fig. 3; range of *R*^2^ and RMSE: 0.884–0.915 and 0.015–0.026, respectively, vs. 0.588–0.721 and 0.035–0.07 for the latter). The variance across 30 trials was also consistently smaller in the former (e.g., standard deviation for *R*^2^ range of 0.018–0.033 vs. 0.034–0.061).

**Figure 4:**
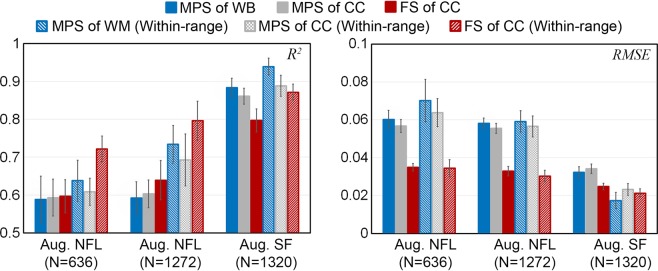
*R*^2^ (left) and RMSE (right) when using three training datasets to test on the same measured SF impact dataset. Increasing the training samples in the augmented NFL dataset improved performances but were still outperformed by those using the augmented SF dataset for training, even when the training sample sizes were comparable. MPS: maximum principal strain; FS: fiber strain; WB: whole brain; CC: corpus callosum.

Figure 4 reports *R*^2^ and RMSE using three augmented datasets for training but the same measured SF dataset for testing. Increasing the augmented NFL training sample size consistently increased *R*^2^ and lowered RMSE (*p* < 0.001), especially for impacts within the targeted rotational velocity peak range. Still, using the augmented SF dataset for training significantly outperformed those using the augmented NFL for training (*p* < 0.001), even when the sample sizes were comparable (e.g., *R*^2^ of 0.871 vs. 0.796, and RMSE of 0.021 vs. 0.03, for within-range fiber strain in the CC).

**Figure 5:**
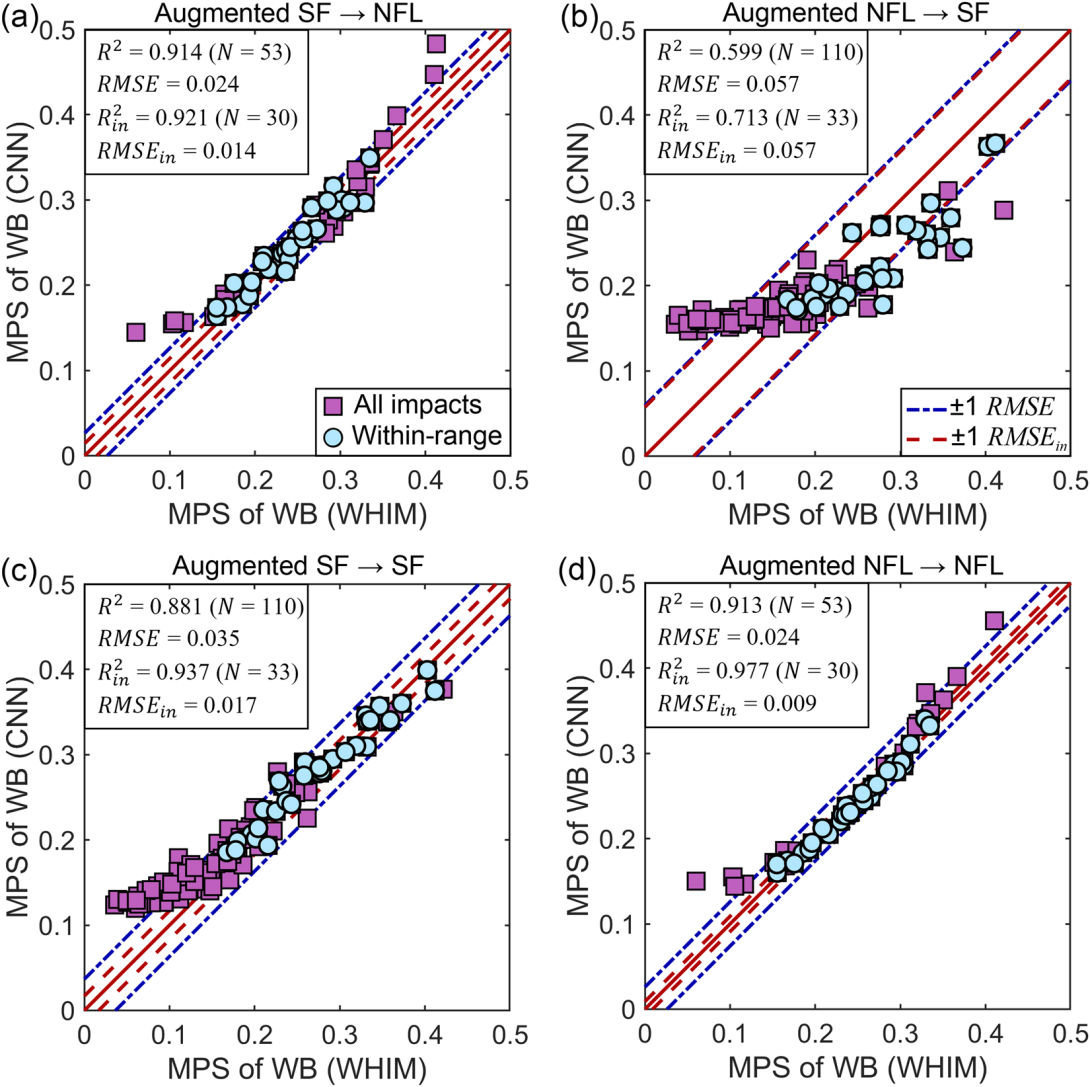
Typical prediction performances for four training-testing configurations (**a**–**d**; augmented NFL dataset used all four batches). Each selected plot reflects a typical trial, with its performance in terms of *R*^2^ closest to the average value from the 30 random trials. Subscript, *in*, refers to impacts within the targeted resultant rotational velocity peak range. MPS: maximum principal strain; WB: whole brain.

Figure 5 compares the predictions with the directly simulated MPS of the whole brain for four training-testing configurations. When the training and testing datasets were from the same source, (Fig. 5c,d), the testing performance was always satisfactory (e.g., *R*^2^ > 0.937 and RMSE < 0.018 for within-range impacts). Using the augmented SF dataset also successfully predicted strains for the reconstructed NFL dataset (*R*^2^ = 0.921 and RMSE = 0.014 for within-range impacts; Fig. 5a). However, when using the augmented NFL dataset to predict measured SF dataset, the performance was notably poorer (e.g., *R*^2^ = 0.599 and RMSE = 0.057 for all impacts; Fig. 5b). For the three “successful” predictions, CNN predictions for out-of-range impacts remained either within the ±1 RMSE range for many (overall *R*^2^ > 0. 880) or somewhat overestimated.

### Performance evaluation: combining the training datasets

**Figure 6:**
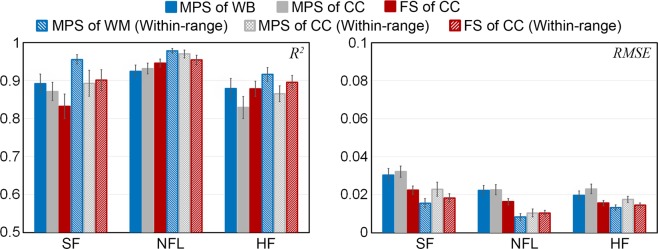
*R*^2^ (left) and RMSE (right) when using the augmented training datasets combined (N = 2592 impacts) to predict brain strains for the measured SF, reconstructed NFL, and measured HF datasets based on 30 random trials, respectively. MPS: maximum principal strain; FS: fiber strain; WB: whole brain; CC: corpus callosum.

Figure 6 reports the testing performances on the three measured impact datasets by combining the augmented SF and NFL datasets for training. Testing on the reconstructed NFL dataset achieved the highest *R*^2^ of 0.978 with RMSE of 0.008 for within-range MPS of the whole brain. Compared to those when using the augmented SF dataset alone for training (Fig. 3), they were increased by 7–9% and decreased by 38–45%, respectively (*p* < 0.01). A smaller performance gain was also observed for the within-range impacts in the measured SF dataset (*R*^2^ increased by 2–4% and RMSE decreased by 1–14% as compared to those when using the augmented SF dataset alone for training; Fig. 3).

**Figure 7:**
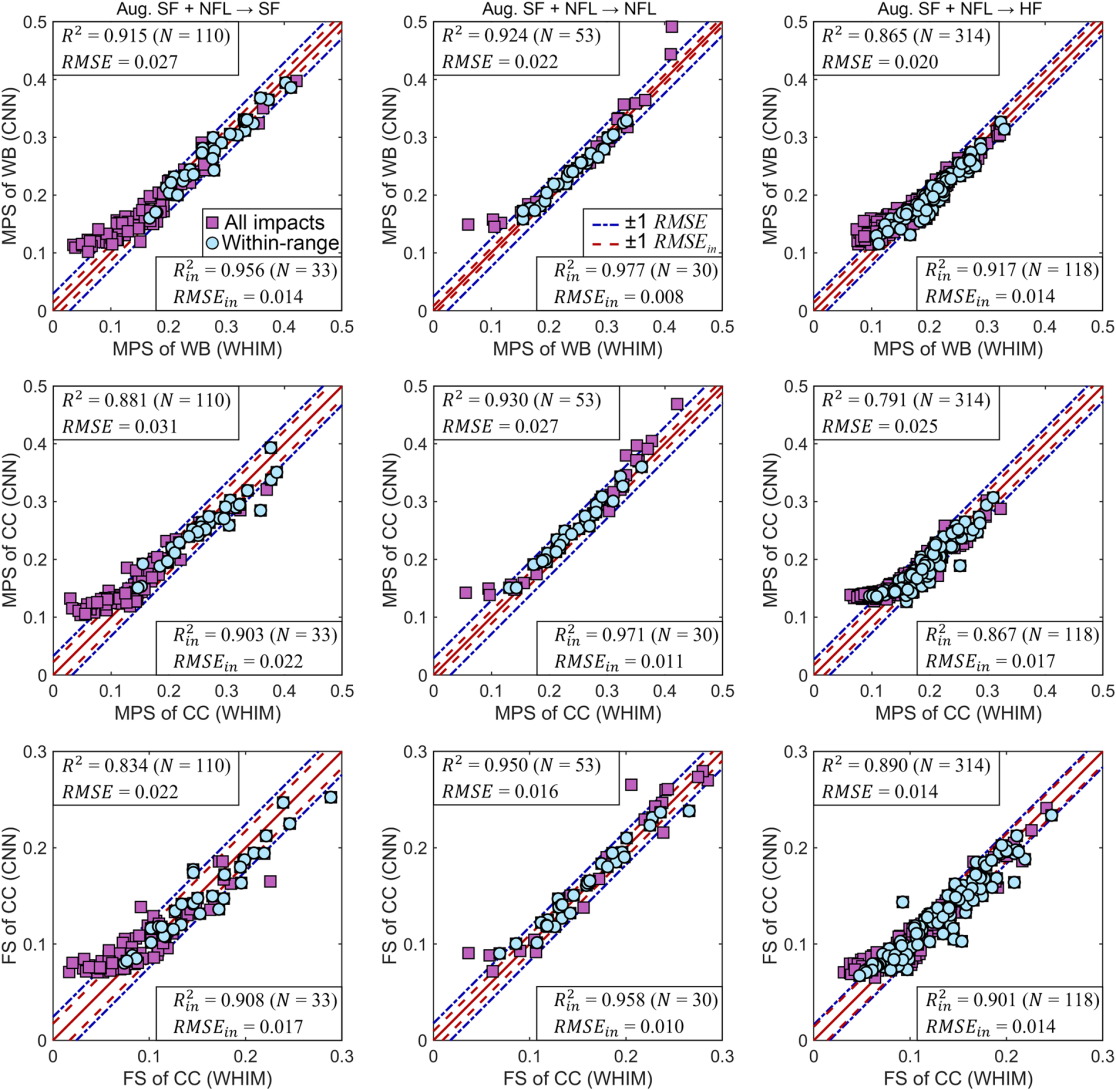
Typical CNN prediction performances for maximum principal strain (MPS) of the whole brain (WB; top), MPS of the corpus callosum (CC; middle row) and fiber strain (FS) of the CC (bottom) for three impact datasets: measured SF (left), reconstructed NFL (middle column), and measured HF (right).

When tested on the third HF dataset, the testing *R*^2^ for MPS of the whole brain achieved the highest value of 0.916, with RMSE consistently <0.02 for within-range impacts. Figure 7 compares the predicted three strain measures with the directly simulated counterparts for the three impact datasets in a typical trial using the augmented datasets combined for training.

**Figure 8:**
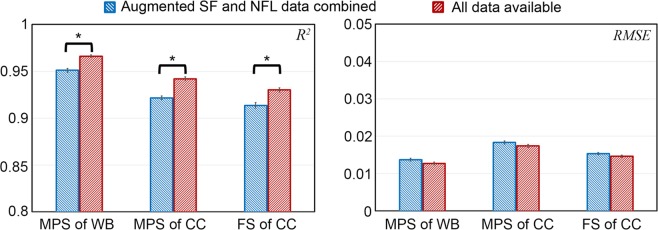
*R*^2^ (left) and RMSE (right) in 10-fold cross-validations using either the augmented training datasets combined (N = 2592) or all impact response data available (N = 3069) based on 30 random trials. MPS: maximum principal strain; FS: fiber strain; WB: whole brain; CC: corpus callosum; “*” indicates *P* < 0.01.

Finally, Fig. 8 compares *R*^2^ and RMSE for the three strain measures in 10-fold cross-validations using either the augmented SF and NFL datasets combined (N = 2592) or all impact response data available (N = 3069). Increasing the training samples significantly improved *R*^2^ (to values of 0.966 ± 0.001, 0.942 ± 0.002 and 0.930 ± 0.002 for MPS of the whole brain, MPS of the CC, and fiber strain of the CC, respectively; *p* < 0.01), but not statistically significant for decreasing RMSE (*p* of 0.051–0.110; RMSE values consistently < 0.018).

## Discussion

Improving the computational efficiency while maintaining the sophistication of a head injury model and accuracy in response output is critical for prospective, real-world applications such as facilitating impact sensors in concussion detection on the sports field. This has long been desired^16,17^ but has remained infeasible to date. In this study, we introduced a data-driven approach using a convolutional neural network (CNN) to learn the nonlinear impact-strain relationship without the need to simplify impact kinematic input^15,28^, head injury model, or response output^19,21,22^. The substantial increase in computational efficiency—from hours on a high-end workstation to under a second on a laptop—may enable a sophisticated head injury model for practical real-world use.

For all training-testing configurations regardless of the strain measure, the trained CNN instantly estimated regional brain strains with sufficient accuracy, especially for within-range impacts. The only exception might be when using the augmented NFL dataset to test on measured SF dataset (Fig. 5b; further discussed below). MPS of the whole brain generally achieved the best performance, with some degradation for more sophisticated strain measures characterizing region-specific MPS and directionally informed fiber strain of the corpus callosum (Figs 7 and 8). Although the regions of interest were limited to the whole brain and corpus callosum for illustration in this study, most likely the technique can be extended to other regions, including specific gray matter regions and their white matter interconnections. In contrast, reduced-order methods are limited to estimating peak MPS of the whole brain^19–22^ but not element- or region-wise MPS, or directionally informed fiber strain in specific regions. The pre-computation technique estimates element-wise MPS of the entire brain^15,28^; however, it remains unclear how it performs when estimating fiber strains.

Nevertheless, directly benchmarking against these competing methods was not feasible in this study because they did not use the same impact data to report performance. Accuracy assessment in this study was limited to those occurring in contact sports; albeit, they were scaled to levels most relevant to injury. In contrast, earlier reduced-order models used impacts from a broad range of head impact conditions including sports, automotive crashes, sled tests and human volunteers^21,22^. However, in addition to degraded accuracy for more severe, but perhaps the most important, impacts, inclusion of low-severity impacts could have also artificially skewed the correlation to a more favorable score. Performance with the pre-computation technique was limited to dummy head impacts, but not real-world recorded impacts yet^28^.

Both the CNN technique and the previous pre-computed brain response atlas approach^15^ require a large training dataset. However, the CNN technique is significantly more advantageous because it is scalable—when model-simulated responses from new or “unseen” impacts become available (e.g., to challenge the CNN estimation accuracy), they can be assimilated into the existing training dataset to re-train. This iterative updating process is expected to continually improve the estimation accuracy, as illustrated (Fig. 8). In contrast, the pre-computation approach is unable to assimilate fresh impact-response samples^15^.

Even for impacts whose resultant peak velocity fell outside of the targeted peak resultant velocity range, many of their CNN predictions were still within ± 1 RMSE relative to their directly simulated counterparts (Figs 5 and 7). This indicated some impressive generalizability and robustness of the CNN technique. The ReLU activation functions were very effective in characterizing the inherently nonlinear brain-skull dynamic system, as it introduced sparsity effect^54^ on the neural network to improve information representation and to avoid overfitting^33^. ReLU was also desirable here since its output and strains of interest were all non-negative. Nevertheless, further fine-tuning the CNN architecture may be desirable in the future using all of the impact-strain response samples available (N = 3069).

The data augmentation scheme *via* permutation and random perturbation (Fig. 1) was important to generate sufficient data variations to the head rotational axis and velocity magnitude. However, the temporal “shapes” of the head rotational velocity profiles were limited to what the measured/reconstructed impact datasets offered. Unfortunately, a parameterized descriptor of head rotational velocity temporal shape does not exist to allow generating its variations for CNN training. However, potentially this could be somewhat augmented by linearly scaling along the temporal direction, which can be explored in the future. Nevertheless, shifting the rotational velocity profiles so that the resultant peak velocity occurred at a fixed temporal location indeed slightly improved the testing performance; otherwise, *R*^2^ typically dropped by 0.01 for within-range evaluations.

We chose to use *v*_*rot*_ profiles for training because *v*_*rot*_ is known to be important to brain strain^15^. The corresponding rotational acceleration profiles or the combination of rotational velocity and acceleration profiles could also be used for training. They are equivalent in prescribing head motion, with the caveat of a possible non-zero initial velocity in *v*_*rot*_ profiles, which was found to be negligible for brain strain. Therefore, they were found to lead to virtually identical CNN performances (confirmed but not shown). The CNN input matrix was fixed to 3 in the first dimension to correspond to the three anatomical directions, but the second temporal dimension could be adjusted. We found that increasing the temporal resolution did not improve CNN estimation accuracy, but decreasing the temporal resolution degraded the accuracy. This suggested that the latter led to some loss of information, as expected.

Comparing performances across different training-testing configurations, we found that training using the augmented SF dataset to test on the reconstructed NFL dataset outperformed that when instead, using the augmented NFL dataset for training to test on the measured SF dataset, even when the sample sizes were comparable (Fig. 5). We suspected that this was because the measured impacts on the field contained more “feature” variations in the rotational kinematics profiles that included acceleration, deceleration, as well as reversal in rotational velocity^8,28^. In contrast, lab-reconstructed head impacts mainly focused on matching impact location, direction, and speed^36^ but may not include more complicated shape variations. These findings suggested the importance of using “feature-rich” impact kinematics data to maximize variation in the training samples. Nevertheless, the addition of the augmented NFL dataset did improve testing performance (Figs 3 vs. 6), suggesting that they also contained information useful for CNN training. On the other hand, prediction on relatively “simple” impacts such as those in the reconstructed NFL dataset always yielded the best performance (Figs 5–7).

Finally, although sophisticated computational hardware is desirable/necessary for model simulations^40^ to generate training samples and to train the CNN, this is not necessary for using an already trained CNN for prediction. In fact, even a portable mobile device may be sufficient for applying a trained CNN for prediction, which is desirable for potential real-world deployment in the future. To potentially better serve the research community and other interested parties, we have made the trained CNN publicly available, along with code and examples at https://github.com/Jilab-biomechanics/CNN-brain-strains. It is anticipated that the link will be updated as needed in the future.

### Limitations

For brevity, we only reported accuracies of strains measured at the 95^th^ percentile for illustration. The same approach was also tested at the 100^th^ and 50^th^ percentile levels (both used in injury prediction). A lower percentile would effectively serve as a smoothing filter to the impact-response hypersurface, which would slightly improve the prediction accuracy compared to the 100^th^ percentile responses (confirmed but not shown).

We focused on a relatively narrow range of peak resultant velocity magnitude because they were most relevant to injury. However, expanding the coverage range to lower impact severities to allow considering the cumulative effects of repeated sub-concussive impacts is straightforward. For out-of-range impacts, the CNN generally overestimated strains to some degree, but with many still within ±1 RMSE relative to the directly simulated counterparts. This highlighted the generalizability and robustness of the technique, which was important for real-world applications where unexpected, “out-of-range” impacts may occur.

Further, the data-driven CNN technique does not address any physics behind brain biomechanical responses. However, as a fast and accurate brain strain response generator, the trained CNN may allow other researchers to efficiently produce impact-response data to explore the physics behind brain strains and concussion in reduced-order models^55^.

Nevertheless, the CNN was only tested using impacts in contact sports in this study because our focus was to enable head injury models for facilitating concussion detection on the sports field. It merits further investigation into whether the application can be expanded more broadly to other impact scenarios such as automotive crashes^21,22^. If found to be similarly effective, the technique may allow transforming state-of-the-art impact kinematics-based studies of brain injury into focusing more on brain strains in the future.

Finally, the trained CNN is model-dependent, and regional strain estimates are subject to all limitations related to the WHIM used for generating training samples. Nonetheless, the CNN can be easily re-trained to accommodate another model or a future, upgraded WHIM. In this case, a large amount of impacts may need to be simulated again. However, existing training data already simulated can still be reused to set an appropriate initial starting point for CNN training, which would reduce the number of impacts required to re-simulate.

## Conclusion

In this study, we developed a deep convolutional neural network (CNN) to train and instantly estimate impact-induced regional brain strains with sufficient accuracy. The technique is significantly more advantageous than other alternative methods, because it does not need to simplify impact kinematic input, head injury model, or response output, and is effective for estimating more sophisticated, region-specific and directionally informed strains. The trained neural network is uniquely capable of assimilating fresh impact-response samples to iteratively improve accuracy. Together with sensors that measure impact kinematics upon head collision, this technique may enable a sophisticated head injury model to produce region-specific brain responses, instantly, potentially even on a portable mobile device. Therefore, this technique may offer clinical diagnostic values to a sophisticated head injury model, e.g., to facilitate head impact sensors in concussion detection *via* a mobile device. This is important to mitigate the millions of concussion incidents worldwide every year. In addition, the technique may transform current acceleration-based injury studies into focusing on regional brain strains.

## References

[1] Peden, M. *et al*. *World report on road traffic injury prevention* (2004).

[2] CDC. *Report to Congress on Traumatic Brain Injury in the United States: Epidemiology and Rehabilitation*, 10.1161/HYPERTENSIONAHA.111.186106 (2015).

[3] Cassidy JD (2004). Incidence, risk factors and prevention of mild traumatic brain injury: results of the WHO Collaborating Centre Task Force on Mild Traumatic Brain Injury. J Rehabil Med 43.

[4] Dompier TP (2015). Incidence of concussion during practice and games in youth, high school, and collegiate American football players. JAMA Pediatr..

[5] Graham, R., Rivara, F. P., Ford, M. A., Spicer, C. M. & Graham, R. *Sports-Related Concussions in Youth*. *Jama* **311** (2014). 10.1001/jama.2013.282985 24185195

[6] Thurman D, Branche C, Sniezek J (1998). The Epidemiology of Sports-Related Traumatic Brain Injuries in the United States: Recent De- velopments. J. Head Trauma Rehabil..

[7] Greenwald RM, Gwin JT, Chu JJ, Crisco JJ (2008). Head Impact Severity Measures for Evaluating Mild Traumatic Brain Injury Risk Exposure. Neurosurgery.

[8] Hernandez F (2015). Six Degree-of-Freedom Measurements of Human Mild Traumatic Brain Injury. Ann. Biomed. Eng..

[9] Bartsch A, Samorezov S, Benzel E, Miele V, Brett D (2014). Validation of an ‘Intelligent Mouthguard’ Single Event Head Impact Dosimeter. Stapp Car Crash J..

[10] Beckwith JG (2013). Head Impact Exposure Sustained by Football Players on Days of Diagnosed Concussion. Med. Sci. Sports Exerc..

[11] King, A. I. A. I., Yang, K. H. K. H., Zhang, L., Hardy, W. N. W. W. N. & Viano, D. C. D. C. Is head injury caused by linear or angular acceleration? in *IRCOBI Conference* 1–12 (2003).

[12] Kleiven S (2007). Predictors for Traumatic Brain Injuries Evaluated through Accident Reconstructions. Stapp Car Crash J..

[13] Mihalik JJP, Lynall RRC, Wasserman EEB, Guskiewicz KMK, Marshall SWS (2017). Evaluating the ‘Threshold Theory’: Can Head Impact Indicators Help?. Med. Sci. Sports Exerc..

[14] Yang KH (2006). Development of numerical models for injury biomechanics research: a review of 50 years of publications in the Stapp Car Crash Conference. Stapp Car Crash J..

[15] Ji S, Zhao W (2015). A Pre-computed Brain Response Atlas for Instantaneous Strain Estimation in Contact Sports. Ann. Biomed. Eng..

[16] Franklyn M, Fildes B, Zhang L, King Y, Sparke L (2005). Analysis of finite element models for head injury investigation: reconstruction of four real-world impacts. Stapp Car Crash J..

[17] Takhounts EG (2008). Investigation of traumatic brain injuries using the next generation of simulated injury monitor (SIMon) finite element head model. Stapp Car Crash J..

[18] Zhao W, Choate B, Ji S (2018). Material properties of the brain in injury-relevant conditions – Experiments and computational modeling. J. Mech. Behav. Biomed. Mater..

[19] Laksari K (2015). Resonance of human brain under head acceleration. J. R. Soc. Interface.

[20] Gabler LF, Joodaki H, Crandall JR, Panzer MB (2018). Development of a Single-Degree-of-Freedom Mechanical Model for Predicting Strain-Based Brain Injury Responses. J. Biomech. Eng..

[21] Gabler Lee F., Crandall Jeff R., Panzer Matthew B. (2018). Development of a Second-Order System for Rapid Estimation of Maximum Brain Strain. Annals of Biomedical Engineering.

[22] Gabler Lee F., Crandall Jeff R., Panzer Matthew B. (2018). Development of a Metric for Predicting Brain Strain Responses Using Head Kinematics. Annals of Biomedical Engineering.

[23] Giordano, C. & Kleiven, S. Evaluation of Axonal Strain as a Predictor for Mild Traumatic Brain Injuries Using Finite Element Modeling. *Stapp Car Crash J*. **November**, 29–61 (2014). 10.4271/2014-22-0002 26192949

[24] Wright RM, Ramesh KT (2012). An axonal strain injury criterion for traumatic brain injury. Biomech. Model. Mechanobiol..

[25] Cloots RJHH, van Dommelen JAWW, Nyberg T, Kleiven S, Geers MGDD (2011). Micromechanics of diffuse axonal injury: influence of axonal orientation and anisotropy. Biomech. Model. Mechanobiol..

[26] Ji S (2015). Group-wise evaluation and comparison of white matter fiber strain and maximum principal strain in sports-related concussion. J. Neurotrauma.

[27] King, A. I., Yang, K. H., Zhang, L., Hardy, W. N. & Viano, D. C. Is head injury caused by linear or angular acceleration? in *Proc*. *IRCOBI Conf* (2003).

[28] Zhao W, Kuo C, Wu L, Camarillo DB, Ji S (2017). Performance evaluation of a pre-computed brain response atlas in dummy head impacts. Ann. Biomed. Eng..

[29] Zhao W, Ji S (2017). Brain strain uncertainty due to shape variation in and simplification of head angular velocity profiles. Biomech. Model. Mechanobiol..

[30] Yamashita R, Nishio M, Do RKG, Togashi K (2018). Convolutional neural networks: an overview and application in radiology. Insights Imaging.

[31] Voulodimos A, Doulamis N, Doulamis A, Protopapadakis E (2018). Deep Learning for Computer Vision: A Brief Review. Comput. Intell. Neurosci..

[32] Ren S, He K, Girshick R, Sun J (2017). Faster R-CNN: Towards Real-Time Object Detection with Region Proposal. Networks. IEEE Trans. Pattern Anal. Mach. Intell..

[33] Günther Johannes, Pilarski Patrick M., Helfrich Gerhard, Shen Hao, Diepold Klaus (2014). First Steps Towards an Intelligent Laser Welding Architecture Using Deep Neural Networks and Reinforcement Learning. Procedia Technology.

[34] Hastie, T., Tibshirani, R. & Friedman, J. *The Elements of Statistical Learning, Data Mining, Inference, and Prediction*. (Springer, 2008).

[35] Zhao W (2019). Regional Brain Injury Vulnerability in Football from Two Finite Element Models of the Human Head. In IRCOBI.

[36] Sanchez Erin J., Gabler Lee F., Good Ann B., Funk James R., Crandall Jeff R., Panzer Matthew B. (2019). A reanalysis of football impact reconstructions for head kinematics and finite element modeling. Clinical Biomechanics.

[37] Pellman EJ (2003). Concussion in professional football: reconstruction of game impacts and injuries. Neurosurgery.

[38] Ji S, Zhao W, Li Z, McAllister TW (2014). Head impact accelerations for brain strain-related responses in contact sports: a model-based investigation. Biomech. Model. Mechanobiol..

[39] Rowson S (2012). Rotational head kinematics in football impacts: an injury risk function for concussion. Ann. Biomed. Eng..

[40] Zhao W, Ji S (2019). White matter anisotropy for impact simulation and response sampling in traumatic brain injury. J. Neurotrauma.

[41] Zhao W, Cai Y, Li Z, Ji S (2017). Injury prediction and vulnerability assessment using strain and susceptibility measures of the deep white matter. Biomech. Model. Mechanobiol..

[42] Hernandez Fidel, Giordano Chiara, Goubran Maged, Parivash Sherveen, Grant Gerald, Zeineh Michael, Camarillo David (2019). Lateral impacts correlate with falx cerebri displacement and corpus callosum trauma in sports-related concussions. Biomechanics and Modeling in Mechanobiology.

[43] Lusch B, Weholt J, Maia PD, Kutz JN (2018). Modeling cognitive deficits following neurodegenerative diseases and traumatic brain injuries with deep convolutional neural networks. Brain Cogn..

[44] Roy, S., Butman, J. A., Chan, L. & Pham, D. L. TBI contusion segmentation from MRI using convolutional neural networks. In *2018 IEEE 15th International Symposium on Biomedical Imaging (ISBI 2018)* 158–162 (IEEE, 2018), 10.1109/ISBI.2018.8363545

[45] Rane L, Ding Z, McGregor AH, Bull AMJ (2019). Deep Learning for Musculoskeletal Force Prediction. Ann. Biomed. Eng..

[46] Hannink J (2017). Sensor-Based Gait Parameter Extraction With Deep Convolutional. Neural Networks. IEEE J. Biomed. Heal. Informatics.

[47] Srivastava N, Hinton G, Krizhevsky A, Sutskever I, Salakhutdinov R (2014). Dropout: A Simple Way to Prevent Neural Networks from Overfitting. J. Mach. Learn. Res..

[48] Nair, V. & Hinton, G. E. Rectified Linear Units Improve Restricted Boltzmann Machines. *Proc. 27th Int. Conf. Mach. Learn*. 807–814, 10.1.1.165.6419 (2010).

[49] Kingma, D. P. & Ba, J. L. Adam: a Method for Stochastic Optimization. *Int. Conf. Learn. Represent. 2015* 1–15, 10.1145/1830483.1830503 (2015).

[50] Chollet, F. & others. Keras (2015).

[51] Prechelt Lutz (1998). Early Stopping - But When?. Lecture Notes in Computer Science.

[52] Bouckaert Remco R., Frank Eibe (2004). Evaluating the Replicability of Significance Tests for Comparing Learning Algorithms. Advances in Knowledge Discovery and Data Mining.

[53] Welch BL (1947). The Generalization of ‘Student’s’ Problem when Several Different Population Variances are Involved. Biometrika.

[54] Glorot, X., Bordes, A. & Bengio, Y. Deep Sparse Rectifier Neural Networks. in *Proceedings of the Fourteenth International Conference on Artificial Intelligence and Statistics* 315–323 (2011).

[55] Abderezaei J (2019). Nonlinear Dynamical Behavior of the Deep White Matter During Head Impact. Phys. Rev. Appl..

